# Clinical Cases

**Published:** 1890-03

**Authors:** G. W. Sherbino

**Affiliations:** Abilene, Texas


### CLINICAL CASES.

G. W. Sherbino, M. D., Abilene, Texas.

Irritable Ulcer of the Anus.—Mr. C., dry goods clerk, has been suffering for some time with painful ulcer of the anus. He had had the diagnosis and treatment of two old school doctors. They cauterized the ulcer. Its location must have been close to the verge of the anus, as he suffered great pain during and after movement of bowels. Two months before he had contracted gonorrhœa, which was cured, in the usual way, by injections.

Subjective Symptoms.—He said of his own accord that the stools were as hot as boiled lead passing through the rectum. Pain after stool lasting for many hours, so that he was in great pain from contraction of the anus. Pain worse from motion, even moving his feet; and yet he got so very restless that he must move. It always made him worse, and he got no relief from it whatever.

I gave him a guarded prognosis, told him he would get discouraged a good many times before he was cured. I thought that his symptoms called for Thuja, and I gave him one dose of 1 M on the tongue. Sac-lac. to last three days. On the third day he called again very much improved. He said he commenced to feel a relief at about seven P. M.—the dose was given at one P. M. He was to report as soon as Sac-lac. had given out. That one dose cured him.

Diarrhœa.—(1) Mrs. E——, sick three or four days from eating green corn. Diarrhœa worse in the morning as soon as she gets up to move around. Gurgling in the centre of the abdomen before the bowels move. Can scarcely retain the stool. Aloes $^{50m}$, one dose. In a few days came back with urine symptoms. One dose Aloes (H. S.)$^{cm}$. Cured for good.

(2) A man, æt. sixty, has had diarrhœa for some time, the trouble is about the same during the day or night.

Subjective Symptoms.—Burning on the vertex. Feet get hot at night, must put them out of bed, cannot sleep with any cover over them they get so hot. Must sleep with the windows and doors open; hungry, all-gone feeling in the stomach about eleven A. M.; weak, faint spells. More in the forenoon.

Before stool terrible griping in the hypogastric region.

Rumbling and gurgling in the left hypochondrium (Aloes, Lycopod., Sanicula).

He has to be very careful about letting flatus pass, as sometimes the stool escapes (Aloes). Vertigo, zigzag, glimmering up and down. This comes on after a feeling in the stomach as though small fry were swimming. A trembling sensation goes up to the head, and the vertigo comes on. It is worse from motion, better when quiet. As the feeling in the stomach subsides the glimmering commences to rise, and ascends higher and higher till it vanishes. ℞ Sulph.$^{55m}$ (Fincke), one dose ; Sac-lac. every half-hour.

Second day, reports slept well all night; had only one stool to-day ; this evening pain all gone; the rumbling and gurgling all gone; feels much better every way, but the glimmering. That has troubled him some this evening ; has hot flashes over each cheek ; a sensation as though he had a fever; it does not last long. ℞ Sac-lac. every two hours.

I came very near making a mistake. The gurgling made me think of Aloes, especially the insecurity when passing flatus; also Lyc., but I could not get the four-o'clock aggravation ; also Sanicula, but the *cold neck* and sweat were absent, so I decided to give Sulph., which cured.

(3) Mrs. McC., æt. twenty-two years, has had diarrhœa for four months. One month ago I attended her in confinement. I made but one visit, and she was weak from having the diarrhœa so long. I gave no medicine at the time of labor, but one month later I was called to see her. She was pale, emaciated very much, could only walk a little around the room. Pulse 120; temperature 100°. The *only* symptom I could obtain was fullness after meals. Gurgling in the left hypochondrium before stools. Exacerbation from four to eight P. M. Lycopodium$^{3m}$ one dose. I called second day. The bowels had moved only once that day. Temperature 100° ; pulse 120; third day, temperature 98 2-5° ; pulse 80. Discharged case. She is now taking on flesh. I had no idea of curing such a severe case. I gave the father a guarded prognosis.

Cholera Morbus.—I was called to see Captain E. He said he had been taken with vomiting and purging that morning about six A. M. Severe *aching in the bones*, and so numb he thought he would be paralyzed. His features were pinched, his voice hoarse and weak. *Hands were numb.* Tongue coated brownish, fetid breath. Temperature 97°. Baptisia$^{cm}$ (Fincke), one dose. Sac-lac. every ten minutes till better. That one dose brought prompt relief and the sick man to health again.

Little boy, aged seven years, was taken at nine P. M. with vomiting and purging. Had eaten nothing all day. Was very thirsty for water. Wanted to drink large quantities at a time, but as soon as it was down he would vomit it up again. Nothing stayed on his stomach. Very restless; burning pain in the stomach, great prostration after vomiting or purging. Gave one dose Arsenicum CM. No more vomiting. His father thinks there must be "something in" my sugar pills.

HEMORRHAGE FROM BOWELS.—Mr. C. L. W., lymphatic temperament, weight two hundred and twenty-five pounds, had just come home from New York. He was taken sick with profuse discharge from the bowels. His wife gave him Acon. first day. Second day she gave him Merc-viv. I found the following symptoms: Pulse 80, strong and full; temperature 99.2°. He passed about half teacupful of blood every twenty minutes. Before the bowels would move he would feel a gurgling in the left side just under the short ribs, then it passed down the descending colon to the rectum, then tenesmus and urging, and his bowels would move (Lycopod.). Bowels very sore across the hypogastric region to touch. Straining during stool. "Feels chilly." *Face dark, flushed, looking as if he had been drinking.* Eyes have a languid look, could with difficulty keep the lids up (Causticum). "*Feels sleepy, could sleep all the time.* Bowels move whenever he moves around (Aloes, Bry.); felt better when quiet, lying down. Baptisia CM (Fincke), one dose, cured; was able to be at the store in twenty-four hours.

CONSTIPATION DURING PREGNANCY.—Mrs. A., eighteen years old, light hair and blue eyes, primipara. Has had constipation ever since she became enciente. I had given her several remedies with no relief whatever.

The fore part or the first of the stools is hard; the after or last part loose or diarrhœic. Itching, burning, and redness of the hands and feet. She says she can tell when she is going to be constipated, as her hands and feet itch and burn so, worse at night. She can scarcely sleep. Agaricus-mus. CM, one dose, made her better for two weeks. One dose more cured.

